# The coffee agroecosystem: bio-economic analysis of coffee berry borer control (*Hypothenemus hampei*)

**DOI:** 10.1038/s41598-020-68989-x

**Published:** 2020-07-23

**Authors:** José Ricardo Cure, Daniel Rodríguez, Andrew Paul Gutierrez, Luigi Ponti

**Affiliations:** 1 Facultad de Ciencias Básicas y Aplicadas, Universidad Militar Nueva Granada, Cr.11 No.101-80, Bogotá, Colombia; 2 Division of Ecosystem Science, College of Natural Resources, University of California, Berkeley, CA 94720-3114 USA; 3 Agenzia nazionale per le nuove tecnologie, l’energia e lo sviluppo economico sostenibile (ENEA), Centro Ricerche Casaccia, Via Anguillarese 301, 00123 Rome, Italy; 4 Center for the Analysis of Sustainable Agricultural Systems Global (Casasglobal.Org), 37 Arlington Ave., Kensington, CA 94707 USA

**Keywords:** Ecological modelling, Environmental economics

## Abstract

Coffee, after petroleum, is the most valuable commodity globally in terms of total value (harvest to coffee cup). Here, our bioeconomic analysis considers the multitude of factors that influence coffee production. The system model used in the analysis incorporates realistic field models based on considerable new field data and models for coffee plant growth and development, the coffee/coffee berry borer (CBB) dynamics in response to coffee berry production and the role of the CBB parasitoids and their interactions in control of CBB. Cultural control of CBB by harvesting, cleanup of abscised fruits, and chemical sprays previously considered are reexamined here to include biopesticides for control of CBB such as entomopathogenic fungi (*Beauveria bassiana*, *Metarhizium anisopliae*) and entomopathogenic nematodes (*Steinernema* sp., *Heterorhabditis*). The bioeconomic analysis estimates the potential of each control tactic singly and in combination for control of CBB. The analysis explains why frequent intensive harvesting of coffee is by far the most effective and economically viable control practice for reducing CBB infestations in Colombia and Brazil.

## Introduction

The coffee trade is, after petroleum, the most valuable commodity globally in terms of total value (harvest to coffee cup)^1,2^, and yet it faces severe issues with pests^3^. The coffee sector had a retail market value of USD 83 billion, providing jobs for 125 million people on 12.5 million farms worldwide, mostly smallholder farms including 22 Low Human Development Countries (LHDCs). In 2017, 70 per cent of total coffee production worth USD 19 billion was exported^4^. The coffee berry borer (CBB) *Hypothenemus hampei* (Coleoptera: Curculionidae: Scolytinae) is the most important pest of coffee worldwide^5,6^ causing immense economic losses in the main production areas of Central and South America, Indonesia, South East Asia ^7,8^, Hawaii^9,10^, and Puerto Rico^11^ where infestation levels are variable but can reach up to 95%^12^.

**Figure 1:**
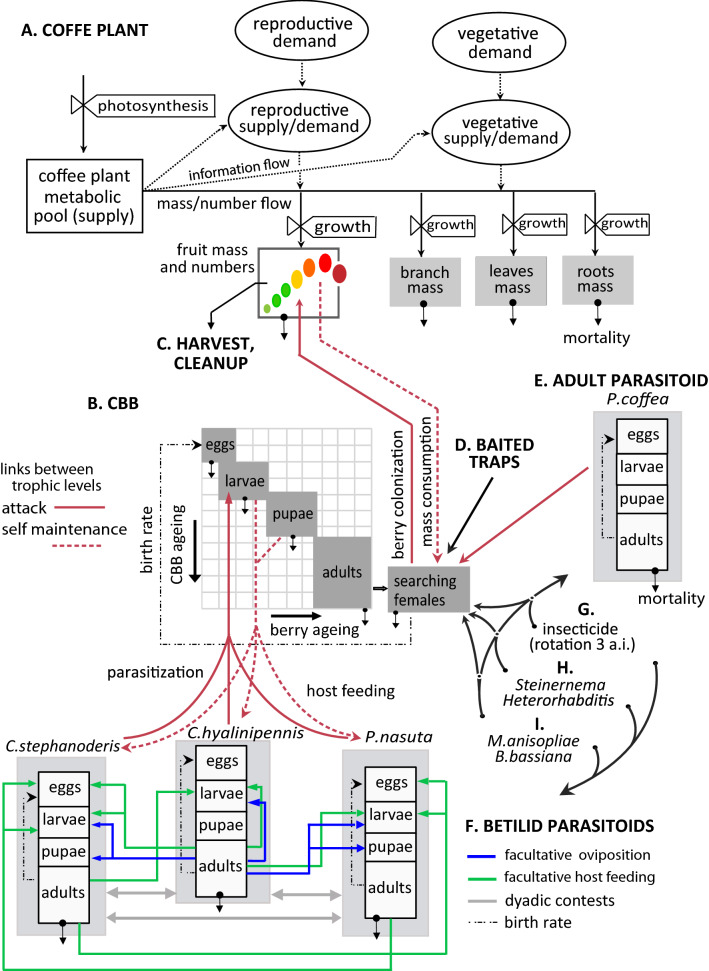
Coffee system with all the components included (modified from Rodriguez et al.^24^). The complete system is embedded in a climate envelope which drives all the development and interaction variables using a PBDM (see text). Diagram of the plant model (**A**) including the link to the CBB model (**B**) and harvest and cleanup as part of CBB cultural management control (**C**). Effect of baited traps on capturing migrant adult females (not included in this paper based on results in Rodríguez et al.^23^) (**D**). The third trophic level is represented by four CBB parasitoids; the adult eulophid parasitoid *Phymastichus coffee* (**E**) and three bethylid parasitoids and their interactions, *Cephalonomia stephanoderis*, *Cephalonomia hyalinipennis* (not included in this paper based on results in Rodríguez et al.^24^) and *Prorops nasuta* (**F**). Three active ingredients (a.i.) of insecticides are include in rotation, affecting the whole system (**G**), as well as the entomopathogenic nematodes *Steinernema* sp. and *Heterorhabditis* sp. (**H**) and the entomopathogenic fungi *Metarhizium anisopliae* and *Beauveria bassiana* (**I**). The dashed line indicates information flow.

Like many ambrosia beetles that form galleries for the development of progeny^13^, mated adult CBB females bore into coffee berries to form galleries. CBB is not effectively controlled by the action of parasitoids^14^, the establishment of which in new world coffee has proven difficult^15–18^. An early multitrophic study of the coffee agroecosystem using the mechanistic physiologically based demographic modeling (PBDM) approach^19,20^ demonstrated that CBB parasitoids alone were not efficient in controlling CBB^21^. Those findings were confirmed in subsequent restudy of the coffee system using PBDMs that introduced extensive new data from Colombia on the dynamics of coffee growth and development^22–24^, the effects of solar radiation on floral bud initiation; effects of leaf water potential on breaking dormancy in flower buds; effects of low temperature on photosynthesis and defoliation; enhanced CBB biology and population dynamics including the effects of intraspecific competition, temperature and rainfall on CBB adult emergence; the impact of baited traps for CBB control; and refinements of parasitoid biology and interactions (i.e. intra and inter competition). These PBDMs provided excellent fits to the field data on coffee growth and development, on CBB dynamics, and provide a solid base for evaluating the efficacy of the four parasitoids singly and in combinations in mitigating the impact of CBB (see review in the supplemental materials). In this paper, we add to the PBDM system the following factors: (1) Conventional cultural practices using intensive harvesting, cleanup of abscised berries, and insecticides, and (2) biopesticides based on two entomopathogenic fungal species (*Beaveria bassiana* and *Metarhizium anisopliae*), and two entomopathogenic nematodes (*Steinernema* sp. and *Heterorhabditis* sp.), and (3) the interaction of all control components. The scope of the coffee system components included in the analysis are depicted in Fig. 1. Field data are time consuming and prohibitively expensive to collect and are unlikely to yield global conclusion across time and geographic space. Realistic mechanistic weather driven PBDMs used as the objective function in our bio-economic analysis are not constrained by such limitations. The control components in the bio-economic analysis are described below.

## Control components

1. Intensive harvesting, cleanup, and insecticide.

There are two main periods of fruit ripening in the Colombian coffee region, April–June, and September–November^25^, but occasional dry periods occur in between that induce the presence of ripe fruits on the plant and abscised fruit on the ground^25,26^. Common cultural practices for CBB control are periodic harvesting of overripe and dry fruits on the tree and cleanup of abscised fruit on the ground (hereafter termed harvest and cleanup respectively)^27–30^.

Insecticides are commonly used for CBB control^31–33^, causing mortality of free-flying adult females before they bore into berries. The action of insecticides decreases over 15 days from the time of application. Benavides and Arévalo^27^ and Benavides et al*.*^32^ recommended, that chemical control should start 90 days after flowering, when berries have a dry matter content of approximately 20%, and that it should be continued if infestation levels are higher than 2%. However, because berries are often present year around in Colombia, it is difficult to apply this criterion for initiating chemical control^25,26^. A further drawback is that the chemicals are hazardous to farm workers and adversely affect biodiversity conservation, particularly natural enemies resulting in outbreaks of new pests^34,35^ and contamination of water and soils^31^, but also pollinators potentially resulting in decreased fruit set^36^. Due to these drawbacks, it has been suggested that farmers should increase resources for field sanitation^37^.

2. Biopesticides of fungal pathogens (*Beaveria bassiana* and *Metarhizium anisopliae*).

Sprays infused with the fungus *B. bassiana* have been reported as an effective control agent of CBB adults^38–44^. Using *B. bassiana* as a stand-alone method of control was questioned by Hollingsworth et al.^45^ The infection rates in the field depend on climatic conditions and strain of the pathogen, with mortality rates ranging from 17.7%^39^ at a concentration of 10^6^ conidia/ml to 40.6%^40^ at a concentration of 10^9^ conidia per plant, though Bustillo^46^ reported mortality as high as 75%. The spores must come in contact with the beetles, and hence are most infective on new adults emerging from host berries or during the initial phases of fruit penetration^47^. Infection by *Metarhizium anisopliae* has a similar etiology, with reported infection rates ranging between 22.1%^40^ and 43.1%^48^.

2. Biopesticides of nematodes (*Steinernema* and *Heterorhabditis*).

Nematodes are not widely employed in commercial crops due to their high cost of production. Experimental evidence in laboratory suggests that they could be useful against CBB stages^49–51^. A field evaluation confirmed that both species can be effective in controlling CBB in abscised fruits in the soil with mortalities of as high as 80% sixty days after the application of 250,000 infective *Steinernema* sp. juveniles per tree. Similarly, applications of 125,000 infective *Heterorhabditis* sp. juveniles per tree can cause 75% mortality after 75 days^52^.

4. The action of CBB parasitoids.

Several studies have reported the use of parasitoids for control of CBB, and their biology were well documented and modeled by Gutierrez et al*.*^21^ and Rodríguez et al*.*^24^. The bethylids *Cephalonomia stephanoderis*^53–58^, *C. hyalinipennis*^18^ and *Prorops nasuta*^53,59–61^ enter the CBB gallery to attack the immature stages. In contrast, the eulophid *Phymastichus coffea*^62–68^ parasitize female CBB adults as they are burrowing into the coffee berry. Results of Rodríguez et al*.*^24^ show that *C. hyalinipennis*, interacts negatively with the other betilids and provides poor control of CBB, and this argues against its introduction, and hence was not considered here. The present work explores only the performance of *C. stephanoderis*, *P. nasuta* and *P. coffea*.

## Methods

### Study sites

Geographical coordinates of the localities and weather data used in these simulation studies are listed in Table 1. The field studies for the plant and CBB were conducted for Colombia (coffee cv. Colombia) during 1990–1995^69^ and 2009–2010^22,23^, and during 1982–1986 for Londrina, PR, Brazil (coffee *cv*. Mundo Novo)^23^. Daily maximum and minimum temperatures (°C), relative humidity (%), precipitation (mm), and hours of sunshine for Colombia were obtained from data published in the Anuario Meterológico Cafetero^70^. Daily solar radiation (MJ∙m^−2^∙day^−1^) was estimated from recorded hours of sunshine using the relationship developed by Prescott^71^. Weather data for Londrina, Brazil were obtained from the Instituto Agronômico do Paraná.

**Table 1:** Summary of the climate data sets from localities included in studies.

Locality	Location	Altitude (m)	Annual rainfall (mm)	Daily average temperature (°C)
**Colombia**				
Buenavista^A^	75° 44′ W 4° 24′ N	1,250	2,060	21.97 ± 1.32**
Chinchiná^B^	75° 39′ W 4° 59′ N	1,400	2,516	21.37 ± 1.21
Marquetalia^C^	75° 00′ W 5° 19′ N	1,450	3,781	20.45 ± 1.24
Ciudad Bolívar^D^	76° 01′ W 5° 51′ N	1,342	2,766	21.53 ± 1.56
**Brazil**				
Londrina^E^	51° 9′ W 23° 18′ S	566	1,622	22 °C ± 1.34

**Mean ± standard deviation.

^A,B,C^Located in the Colombian traditional growing area, planted with *cv*. Colombia (see Riaño et al.^69^).

^D^Located in the middle of a 17 ha coffee plantation in Colombian Central growing area (Antioquia) *cv*. Colombia, three 1 ha sampling pots were stablished (see Rodríguez et al.^24^). ^E^A one ha plot of coffee *cv.* mundo novo in Paraná Brazil at the Instituto Agronomico do Paraná (see Gutierrez et al.^21^).

### Exploring alternatives for CBB management using the PBDM system

#### Cultural control

Simulated harvests (symbol *H*) and cleanup (*CU*) at three different time-intervals (*T* = 15 days, 30 days, and 60 days) were explored. Because the PBDM tracks the age structure of developing fruit, estimates of field harvest efficiency of fruit stages per branch reported by Baker^72^ were used in the model: 8.8% for unripe fruit, 67.3% for ripening fruit, 88.5% for ripe fruit and 53.4% for dry fruit, with harvest efficiency decreasing with tree age (i.e. 87% on 2-year old trees and 62% for 3- and 4-year trees). For cleanups, an efficiency of 53.4% was assumed^72^.

#### Chemical control

Mortality associated with insecticide sprays (*C*) occurs only to free-living adults, both CBB and parasitoids, as all immature stages and not free-living adults are inside fruits and are unaffected^31,33,46^. The active ingredients (*ai* = 1…3) of Chlorpyrifos, Fenitrothion and Phenthoate have been reported as effective for control of CBB females colonizing fruit with  maximum mortalities γ_ai_ = 0.93, 0.95 and 0.97, respectively^33^. Same mortality values were used for the parasitoid *P. coffea*. The realized mortality rate (${\mu }_{ai}$) of CBB and all parasitoids is assumed to decrease exponentially through time (*t*) in days from initial maximum mortality values ($\gamma_{ai}$) , Eq. (1):

1 $$\mu_{ai} = \gamma_{ai} e^{ - \,0.1\,t}$$

The effect of Chlorpyrifos and Fenitrothion on the parasitoid *P. nasuta* was studied by Mejía et al.^73^ and provided estimates for $\gamma$ of 0.96 and 0.97 respectively. These values were also used for the effect of the chemicals on free living *C. stephanoderis*. Data evaluating the effect of Phenthoate on the bethylids were not available, and hence the same value of $\gamma$ for CBB (0.97) was assumed. In the simulations, a rotation scheme of the three sprays was used. The recorded 13 spray dates coinciding with simulated periods of high CBB female emergence and were used for the 5-year simulations for Colombia. Seven spray dates were used for Brazil during the 3-year study.

#### Entomopathogenic fungi

Logistic models assuming a logit link function^74^ were fit to data reported by De la Rosa et al*.*^40^ on the average proportion infection (mortality) by *B*. *bassiana* (strain Bb25), Eq. (2), and *M. anisopliae* (strain Ma4) Eq. (3), on cumulative daily degree days after spraying (i.e.* dda*) and daily relative humidity (*RH*)*.* Daily degree days were computed with a nonlinear model^75^ using maximum and minimum temperatures and lower and upper thermal thresholds of 8 °C and 35 °C respectively for *B. bassiana *(*BB*)^76^, and 11 °C and 35 °C for *M. anisopliae *(*MA*)^77^. Several significant digits are given so the results can be accurately reproduced.

2 $$\mu_{BB} = \frac{{e^{{ - 24.19\,\, + \,\;4.225\,\,{\text{x}}\,10^{ - 3} \,dda^{\;} \, - \,\,{4}{\text{.51}}\,\,{\text{x}}\,10^{ - 6}\, dda^{2}\, + \,\,25.28\,\,RH\,}} }}{{1 + e^{{ - 24.19\,\, + \,\;4.225\,\,{\text{x}}\,10^{ - 3} \,dda^{\;} \, - \,\,{4}{\text{.51}}\,\,{\text{x}}10^{ - 6}\, dda^{2}\, + \,\,25.28\,\,RH\,}} }}$$

3 $$\mu_{MA} = \frac{{e^{{ - 28.30{\kern 1pt} \, + \,\;4.591\,\,{\text{x}}\,10^{ - 3} \,dda^{2} \, - \,\,{5}{\text{.672}}\,{\text{x}}\,10^{ - 6} \,dda^{2}\, + \,\,29.38\,\,RH\,}} }}{{1 + e^{{ - 28.30{\kern 1pt} \, + \,\;4.591\,\,{\text{x}}\,10^{ - 3} \,dda^{2} \, - \,\,{5}{\text{.672}}\,{\text{x}}\,10^{ - 6}\, dda^{2}\, + \,\,29.38\,\,RH\,}} }}\,$$

${\mu}_{BB}$ and ${\mu }_{MA}$ are the proportion infection (i.e. mortality rate) of CBB adult females seeking or starting to colonize new fruits. The pathogens also affect the parasitoids, but data are available only for bethylid *C. stephanoderis* adults, and hence the same function was assumed for *P. nasuta.* Daily mortality rates for *B. bassiana* on parasitoids *P. nasuta*^73^, *C. stephanoderis*^78^ and *P. coffea*^79^ were estimated as a linear function of pathogen (*dda*). Infection and mortality of all developmental stages of *P. coffea* attacking CBB females was assumed^79^.

#### Entomopathogenic nematodes

Infection by nematodes is restricted to CBB in attacked berries and in abscised berries on the ground. Logit functions of infection with an application of 250,000 infective juveniles of *Steinernema* sp. (${{\mu }}_{S}$), Eq. (4) and 125,000 infective juveniles of *Heterorhabditis* sp. (${{\mu }}_{H}$), Eq. (5), were fit to data reported by Lara et al*.*^52^ as functions of cumulative daily degree days after spraying (*dda*) above 8 °C for *Steinernema* and 11 °C for *Heterorhabditis* starting from the beginning of the infection, Eqs. (4) and (5).

4 $$\mu_{S} = \frac{{e^{{\,5.176\; - {\kern 1pt} \,0.1035\,\,dda\, + \,\,{4}{\text{.203}}\,{\text{x}}10^{ - 4} \,dda^{2} - \,\,{6}{\text{.166}}\,\,{\text{x}}\,10^{ - 7} \,dda^{3} + \,{ 3}{\text{.001}}\,{\text{x}}10^{ - 10}\, dda^{4} \,}} }}{{1 + e^{{\,5.176\; - {\kern 1pt} \,0.1035\,\,dda\, + \,\,{4}{\text{.203}}\,\,{\text{x}}10^{ - 4}\, dda^{2} - \,\,{6}{\text{.166}}\,{\text{x}}10^{ - 7}\, dda^{3} +\, { 3}{\text{.001}}\,\,{\text{x}}10^{ - 10} \,dda^{4} \,}} }}$$

5 $$\mu_{H} = \frac{{e^{{\,6.659\; - {\kern 1pt} \,0.09015\,\,dda\, - \,\,{2}{\text{.172}}\,{\text{x}}10^{ - 4} \, dda^{2}\, - \,\,{1}{\text{.429}}\,\,{\text{x}}\,10^{ - 7} \, dda^{3} \,}} }}{{1 + e^{{\,6.659\; - {\kern 1pt} \,0.09015\,\,dda\, - \,\,{2}{\text{.172}}\,{\text{x}}10^{ - 4} \, dda^{2}\, - \,\,{1}{\text{.429}}\,\,{\text{x}}\,10^{ - 7}\, dda^{3} \,}} }}$$

Humidity and ultraviolet radiation may also affect nematode survival and efficacy^80^, but data were not available on these effects and hence were not included in the analysis.

### Data analysis

#### Combination and interactions of control components

The models for the different control factors are modular, and individual factors (independent variables) could be included in simulation runs using Boolean dummy variables (include = 1, exclude = 0), while the simulated cumulative number of CBB infested berries year^−1^ (*I*) was the dependent variable.

Two studies were done. In the first study, the ten combinations of cultural controls (*H, CU*) × time interval treatments widely used by coffee farmers^81^ (Table 2) and the use of chemical insecticides were analyzed. In the second study, 2,560 combinations of control factors were included: cultural control and times, insecticides, the four entomopathogen biopesticides and three parasitoids (*C. stephanoderis*, *P. nasuta* and *P. coffea*).

**Table 2:** Combinations of cultural control strategies.

Combination	*H*	*T *(harvest)	*CU*	*T *(cleanup)
1	0	0	0	0
2	0	0	1	15
3	0	0	1	30
4	0	0	1	60
5	1	15	0	0
6	1	30	0	0
7	1	60	0	0
8	1	15	1	15
9	1	30	1	30
10	1	60	1	60

*H* harvest, *CU* cleanup, *T* time between cultural practices (harvest or cleanup). Note that *T* is shown only for clarity.

Negative binomial regression models assuming a log link function were used to summarize the simulation results, Eq. ($$6)$$. Specifically, log_e_ cumulative number of CBB infested berries⋅year^-1^ (log_e_ *I*) was regressed on presence-absence values of the independent control variables *x*_*i*_*.*

6 $$\log_{e} I = f(x_{1} ,.....,x_{n} ) = a + b_{\,1} x_{1} + ..... + b_{n} x_{n}$$

The log_e_-linear model, Eq. (6), accounts for over dispersion and skewness and satisfies the assumptions of the parametric analysis^82^. The final model was selected using Akaike’s information criteria^83^ retaining only significant independent variables and interactions (p < 0.05). One model was fit to the combined data for the four Colombia localities and another for the single Brazilian locality.

To estimate the magnitude and direction of the impact of a management variable on CBB infestations, the derivative of Eq. (6) was with respects to *x*_*i*_ , Eq. (7).

7 $$\frac{{\partial \log_{e} I}}{{\partial x_{i} }}, \, i = 1,...,n$$

This yields the log_e_ rate of change of infested berries given the action of *x*_*i*_ and the average effects of the other independent variables. Taking the antilog of Eq. (7) we get the infestation rate as a proportion after the action of *x*_*i*_.

## Results

### Analysis of cultural and chemical controls

#### Colombian study

Results of the multiple regression model for cultural controls are summarized in Table 3. If *H* = harvest, *CU* = cleanup, *C* = chemical control and *T* = time is in days between cultural controls, then the marginal log_e_ contributions of each factor, Eq. (7), in reducing *I* are: *H*(${{\partial \log_{e} I} \mathord{\left/ {\vphantom {{\partial \log_{e} I} {\partial H}}} \right. \kern-\nulldelimiterspace} {\partial H}} = - 2.0942$) > *CU*($= - 0.1641$) > *C*($= - 0.1064$), and show that harvesting has the greatest effect in reducing infestation levels. Note that, as time between harvests increases, infestation levels increase, as suggested by the positive marginal contributions for *T* ($= 0.1929$). The same notation (e.g., *H(value)*) will be used in the other sections for other control factors.

**Table 3:** Regression model parameters including control strategies widely used by farmers in Colombia.

Variable	Mean	Regression coefficient	Std. error	p*
Intercept	–	11.5681	0.3147	< 2e−16
*H*	0.6	-2.1767	0.1406	< 2e−16
*T*	33	0.0058	0.003	0.0575
*CU*	0.6	-0.2735	0.0754	0.0002
*C*	0.5	-0.2128	0.0688	0.0002
*H*ˑ*T*	21	0.0025	0.0038	1.03e-10
${log}_{e}$ *I*	10.2354			

*H* Harvest, *T* time between cultural practices (harvest or cleanup), *CU* cleanup, *C* Chemical control, ${log}_{e}$ *I* = predicted value (${log}_{e}$ of CBB infested berries⋅year^−1^) using the mean values of the independent variables.

*Only significant independent variables and interactions are listed (p < 0.05).

The antilog of marginal contribution of each factor (left super script *A*) is the proportion of fruit infested by CBB after the action of the factor given the average effect of the other independent variables: $^{A} H = e^{( - 2.0842)} = 0.1231$, $^{A} CU = e^{( - 0.1641)} = 0.8486$ and $^{A} C = e^{( - 0.1064)} = 0.899$, which again highlights the importance of harvesting over cleanup and chemical control, suggesting that the impact of ^*A*^*H* is 6.9 fold higher than *CU* (i.e., ^*A*^*CU/*^*A*^*H*) and 7.3 higher than ^*A*^*C*.

#### Brazilian study

In Brazil, periodic harvest (*H*) and time interval (*T*) were the only significant factors in the regression model (Table 4). The marginal log_*e*_ contribution of harvest in reducing CBB infestation is *H* (− 2.0343), with the average proportion of berries infested by CBB being $^{A} H = e^{( - 2.0343)} = 0.1307$. As in Colombia, the effect of *T* is positive, indicating that infestation levels increase as the time between treatments increases. Despite the very different growth forms of coffee in Brazil, the values are similar to those estimated for Colombia.

**Table 4:** Regression model parameters including control strategies widely used by farmers in Brazil.

Variable	Mean	Regression coefficient	Std. Error	p*
Intercept		11.9794	0.1687	< 2e−16
*H*	0.6	− 3.709	0.2079	< 2e−16
*T*	33	0.0003081	0.00444	0.945
*H*ˑ*T*	21	0.0579	0.005692	< 2e−16
${log}_{e}$ *I*	9.754			

*H* Harvest, *T* time between harvests, ${log}_{e}$ *I* = predicted value (log_*e*_ of CBB infested berries⋅year^−1^) using the mean values of the independent variables.

*Only significant independent variables and interactions are listed (p < 0.05).

### Analysis of all factors and interactions

#### Colombian study

The regression model of log_*e*_* I* on all independent variables and their combinations (Table 5) shows that all control variables singly reduce infestation levels (negative sign of the coefficient). On the other hand, positive signs for most interaction terms (except *H*ˑ*Pc*ˑ*Bb*) suggest the interactions increase infestation levels due to antagonistic effects among control components. Again, the positive sign of the regression coefficient for time interval *T* indicates that infestation levels increase with increasing time between implementation of cultural control practices. Harvest and cleanup reduce infestation to 13.48% 15 day intervals, but this percentage increases to 18.86% when the interval increases to 30 days, and rises markedly to 37.49% when 60 days.

**Table 5:** Regression model parameters with all variables and combinations for Colombia.

Variable	Mean	Regression coefficient	Std. error	p*
(Intercept)		11.0481	0.1714	< 2e−16
*Cs*	0.5	− 0.0257	0.0055	2.85e−06
*Pn*	0.5	− 0.0286	0.0087	0.000966
*H*	0.6	− 1.5835	0.0161	< 2E−16
*Pc*	0.5	− 0.3751	0.0160	< 2E−16
*Bb*	0.5	− 0.3178	0.0150	< 2e−16
*CU*	0.6	− 0.7607	0.0140	< 2E−16
*C*	0.5	− 0.1381	0.0078	< 2E−16
*Ma*	0.5	− 0.0420	0.0055	1.94E−14
*Het*	0.5	− 0.1388	0.0055	< 2e−16
*Stei*	0.5	− 0.1215	0.0055	< 2e−16
*T*	33	0.0070	0.0004	< 2e−16
*Pn*ˑ*H*	0.3	0.0285	0.0112	0.010793
*Pc*ˑ*Bb*	0.25	0.2960	0.0173	< 2e−16
*Pc*ˑ*H*	0.3	0.1864	0.0161	< 2e−16
*Bb*ˑ*H*	0.3	0.1514	0.0161	< 2e−16
*Pc*ˑ*CU*	0.3	0.0385	0.0116	0.000872
*Pc*ˑ*C*	0.25	0.1580	0.0110	< 2e−16
*Bb*ˑ*CU*	0.3	0.0665	0.0116	8.85e-09
*H*ˑ*T*	21	0.0123	0.0003	< 2e−16
*CU*ˑ*T*	21	0.0034	0.0003	< 2e−16
*H*ˑ*Pc*ˑ*Bb*	0.15	− 0.2084	0.0224	< 2e–16
${log}_{e}$ *I*	9.8322246			

*Cs*, *Cephalonomia stephanoderis*; *Pn*, *Prorops nasuta*; *H*, Harvest; *Pc*, *Phymastichus coffea*; *CU*, cleanup; *T*, time between cultural practices (harvest or cleanup); *C*, chemical control;* Bb*, *Beauveria bassiana*; *Ma*, *Metarhizium anisopliae*; *St*, *Steinernema* sp.; *Ht*, *Heterorhabditis* sp; ${log}_{e}$ *I*, predicted value (${log}_{e}$ of CBB infested berries⋅year^−1^) using the mean values of the independent variables.

*Only significant variables and interactions are included (p < 0.05).

The average marginal log_e_ contributions of the various factors in decreasing CBB infestation (*I*) are: *H*(− 1.0454) > *CU*(− 0.6228) > *Het*(− 0.1388) > *Stei*(− 0.1215) > *Bb*(− 0.1016) > *Pc* (− 0.07564) > *C*(− 0.05907) > *Ma*(− 0.04196) > *Cs*(− 0.02566) > *Pn*(− 0.01148).

These results expressed as average proportion berry infestation rates (antilog) given the average effect of the other independent variables are:

^*A*^*H* = ${e}^{-1.0454}=0.3515$, ^*A*^*CU* $=0.5364$, ^*A*^*Het* $=0.8703$, ^*A*^*Stei* $=0.8856$, ^*A*^*Bb* $=0.9034$, ^*A*^*Pc* $=0.9271$, ^*A*^*C* $=0.9426$, ^*A*^*Ma* $=0.9589$, ^*A*^*Cs* $=0.9746$ and ^*A*^*Pn* $=0.9885$). These results reinforce the notion that frequent harvesting (^*A*^*H*) is the most effective tactic for reducing log_e_ *I* with an effect 1.5-fold > *CU,* 2.47-fold > *Het*, 2.51-fold > *Stei*, 2.57-fold > *Bb*, 2.63-fold > *Pc*, 2.68-fold > *C*, 2.72-fold > *Ma*, 2.77-fold > *Cs* and 2.81-fold > *Pn*.

#### Brazilian study

In Brazil, only a few single variables were significant contributors to the reduction of log_e_ *I* (Table 6), and only the interactions *PcˑC* and *HˑT* had significant positive effects. Contrary to that obtained for Colombia, the interaction *BbˑH* has a negative effect on infestation levels. As in Colombia, the time interval (*T)* between cultural control practices was significant positive. The estimated infestation is 3.59% when the harvest and cleanup interval is 15 days, 9.69% for 30 days and 70.41% for 60 days.

**Table 6:** Regression model parameters with all variables and combinations for Brazil.

Variable	Mean	Estimate	Std. Error	z value	p*
(Intercept)		11.633713	0.0171672	677.671	< 2e−16
*Cs*	0.5	− 0.0363631	0.0079498	− 4.574	4.78e−06
*Pc*	0.5	− 0.1199346	0.0112427	− 10.668	< 2e-16
*C*	0.5	− 0.0859333	0.0112425	− 7.644	2.11e−14
*Bb*	0.5	− 0.0954134	0.0125624	− 7.595	3.07e−14
*H*	0.6	− 4.3176702	0.0181429	− 237.981	< 2e−16
*Ma*	0.5	− 0.1249867	0.0079498	− 15.722	< 2e−16
*Het*	0.5	− 0.0782117	0.0079498	− 9.838	< 2e−16
*Stei*	0.5	− 0.0737914	0.0079498	− 9.282	< 2e−16
*T*	33	0.0015787	0.0003419	4.617	3.89e−06
*Pc*ˑ*C*	0.25	0.1090624	0.0158997	6.859	6.92e−12
*Bb*ˑ*H*	0.3	− 0.1718035	0.0162244	− 10.589	< 2e−16
*H*ˑ*T*	21	0.0645351	0.0004384	147.212	< 2e−16
${log}_{e}$ *I*	10.1188525				

*Cs*, *Cephalonomia stephanoderis*; *Pn*, *Prorops nasuta*; *H*, Harvest; *Pc*, *Phymastichus coffea*; *CU*, cleanup; *T*, time between cultural practices (harvest or cleanup); *C*, chemical control; *Bb*, *Beauveria bassiana*; *Ma*, *Metarhizium anisopliae*; *St*, *Steinernema* sp.; *Ht*, *Heterorhabditis* sp.; ${log}_{e}$ *I*, predicted value (log of CBB infested berries⋅year^−1^) using the mean values of the independent variables.

*Only significant variables are included (p < 0.05).

Average marginal log_e_ contribution to the reduction of *I* are as follows: *H* (− 2.1880) > *Bb* (− 0.1985) > *Ma* (− 0.1250) > *Het* (− 0.07821) > *Stei* (− 0.07379) > *Pc* (− 0.06540) > *Cs* (− 0.03636) > *C*(− 0.03140). The results expressed as average proportion of berries infested are: ^*A*^*H*(${e}^{-2.1880}=0.1121$), ^*A*^*Bb*(${e}^{-0.1985}=0.8200$), ^*A*^*Ma*(${e}^{-0.1250}=0.8825$), ^*A*^*Het*($=0.9248$), ^*A*^*Stei*($=0.9288$), ^*A*^*Pc*($=0.9367$), ^*A*^*Cs*($=0.9643$) and ^*A*^*C*( $=0.9690$).

The order of impact of the control practices differ in some cases from the results for Colombia, although harvest (^*A*^*H*) is again the most effective control method to reduce infestations, with its affect being 7.3-fold > *Bb*, 7.9-fold > *Ma*, 8.2-fold > *Het*, 8.3-fold > *Stei*, 8.3-fold > *Pc*, 8.6-fold > *Cs* and 8.4-fold > *C.*

## Discussion

Bioeconomics is the study of the economics of renewable resource acquisition and allocation applicable to all trophic levels. In human economies, harvesting of renewable resources occur via the economic system^84^. Econometric marginal analysis is best done with extensive field data^85^, but such data may be difficult to collect and is prohibitively expensive (e.g., coffee). However, simulation data generated by a well parameterized, field-validated mechanistic models can provide a highly suitable alternative because the results can be compared to limited field data^86,87^. Our PBDM system developed to simulate the growth and development of coffee, the dynamics and infestation levels of coffee berry borer (CBB) and the action of four parasitoids of CBB is based on extensive data^21–24^ and provide a very suitable platform for including the effects of cultural practices such as harvest (*H*), cleanup of abscised berries (*CU*) and the time intervals between these activities, and the effects of sprays of insecticide and of biopesticides of two fungal pathogens and two nematode parasites.

Intensive international efforts to achieve biological control of CBB have failed. Our simulation results for Colombia explain the average reduction of < 15% by parasitoids, entomopathogens, and chemical control. Additionally, antagonistic effects among these control tactics were found. For example, harvesting and cleanup affected the action of CBB parasitoids, *P. nasuta* (*Pn*) and *P. coffea* (*Pc*) increasing CBB infestation levels. Specifically, positive coefficients for the interactions *PnˑH*, *PcˑH* and *Pc*ˑ*CU* indicate detrimental effects to parasitoid efficacy of harvesting and cleanup because parasitoid life stages are also removed from the system, resulting in lower future CBB parasitization rates. The incompatibility between cultural control and parasitoids was also found by Gutiérrez et al.^21^ and Aristizábal et al.^88^.

Similar antagonistic effects were found for harvests and cleanup with sprays of pathogenic fungi (*B. bassiana*, *Bb*) and cultural practices as indicated by the positive sign interactions of *BbˑH* and *BbˑCU*. Bustillo^47^ reports that sustained efficacy of the pathogen *B. bassiana* in the field is strongly associated with the production of spores from field infected CBB, but harvest and cleanup remove these inoculum sources. Another significant antagonistic effect identified for Colombia was the interaction of the eulophid parasitoid *P. coffea* and *B. bassiana* (i.e. *PcˑBb*). In laboratory studies, Castillo et al.^79^ found that exposure to *B. bassiana* caused mortality rates of 100% in *P. coffea* immature stages and a reduction of 22% in adult longevity which reduces parasitoid efficacy. Chemical control (*C*) also affects the efficacy of *P. coffea* as indicated by the positive interaction *PcˑC*. This occurs because unlike the bethylid parasitoids that enter the berry, *P. coffea* female are entirely free living and attacks CBB females initiating penetration of coffee berries making them susceptible to insecticides^89^.

Despite some detrimental effects on biological control agents, periodic harvest of fruit and clean up were found to be the major control practice reducing CBB infestation levels (*I*) in both Colombia and Brazil, with the efficacy of the practice decreasing as the time (*T*) between harvests (*H*) and cleanup (*CU*) increased from 15 to 60 days. The analysis for Colombia suggests that cleanup is the second most important control strategy for reducing the level of infestations. These simulation result agrees with Johnson et al.^90^, who found that ground and tree raisins (dry overripe fruit) left after harvest, could be the main CBB reservoir in the inter-crop season in Hawaii. The results for these cultural practices also agree with field studies of Duque and Cháves^91^ who found that > 94% of Colombian farmers participating in a survey considered cultural control to be the most important method for reducing CBB populations. Bustillo et al.^92^ found that periodic harvesting reduced CBB populations up to 80%, with Benavides et al.^32^ and Aristizábal et al.^29^ in Colombia and Aristizábal in Hawaii^30^, reporting that periodic harvests at 15 day was the main method for reducing CBB populations, and for generating higher yield and income. Unfortunately, producers have a checkered record of implementation cultural control tactics, as Aristizábal et al.^93^ found that only 45% were applying periodic harvest according to the criteria proposed by Bustillo et al.^92^.

Gutierrez et al.^21^ found for Brazil that harvesting and cleanup (only twice a year) had little impact on control because at harvest most berries were infested, the females inside fruits were near the end of their reproductive life, and most adult progeny had emerged. However, as in Colombia, harvesting was the most important factor reducing CBB infestation.

In summary, harvesting and cleanup at 15-day intervals is the only control tactic that significantly reduces CBB infestation level in Colombia and Brazil. Aristizabal et al.^30^ analyzing the cost of harvesting and cleanups (“sanitation picks") in Hawaii, remarked that while initially the cost appears to be high, in the final analysis, sanitation pays the cost of labor and processing, while reducing the source of the pest. That study for Hawaii and the study of Benavides et al.^32^ for Colombia, shows that harvesting and cleanup can be economically feasible. However, it may not be economic in Londrina, Brazil which is at the southern climatic limits of coffee production, with short dry periods followed by short periods of rain throughout the year, resulting in the production of susceptible berries over a longer period than in Colombia. This fruiting phenology has a strong impact on the dynamics of the system and on CBB control as shown by our PBDM results^22,23^.

The socio-economic conditions differ in various coffee growing regions, the fluctuation of prices in the international market can vary widely (including for premium quality coffee), and infestation levels have an important impact on coffee yield and price^94^. Hence, in economic analyses, control tactics must enter not only as cost, but also as price enhancing attributes. For example, effective CBB control based on sustainable periodic harvesting could be an important element in promoting and positioning select coffees on the international markets as unique, organic, and highest quality. To this end, an in-farm mixture of shade grown, and sun grown coffees using organic cultural practices to control CBB has been proposed as a sustainable option for coffee production on small to medium properties^95^.

In conclusion, our model is a realistic virtual crop system that provides a very useful general tool for investigating aspects not readily amenable to field experimentation and has the capacity to integrate more layers such as a socio-economic one. This tool can also be used to examine new technological opportunities prior to their wide adoption. For example, CBB control may be affected by disrupting the symbiotic bacteria in CBB’s microbiome responsible for caffeine breakdown^96^. Another tactic is the development of attractants that are more competitive with the activeness of coffee berries^96,97^; a tactic that could be especially important because coffee flowering phenology varies widely throughout the world in response to regional climate patterns that influences the phenology and dynamics of CBB infestation and the success of progeny development.

Climate change, including climate variability, must be considered as this may change extant regional dynamics of both coffee and CBB, and their interactions. Increased temperature may generate conditions favorable (or unfavorable) for coffee and CBB allowing range extensions to new areas, and changes in CBB damage levels in its current geographical distribution. Increased dry “El Niño” climatic events in some countries could increase CBB populations, while “La Niña” events with prolonged wet seasons would limit CBB populations. The effects of such phenomena differ across geographical region^7^, and the coffee/coffee berry borer system model provides a framework for analyzing the potential effect of variation in weather, climates and of climate change on coffee yield, and the dynamics of CBB across diverse bio-geographical zones^23^.

As an aside, the high pest status of this species in monocultures is a consequence of an evolutionary background, similar to what have been observed in other systems (e.g., between fruiting in sylvan cotton and cotton weevil, *Anthonomus grandis* Boh.^20^). From the prospective of the ecological theory, the large female bias appears to have had high adaptive value in the African tropical forest where it evolved so that large numbers of the small females with low searching rates could find scattered patches of suitable age berries^23^. This adaptation would appear to occur at the expense of reduction in genetic variability caused by sib-mating and reported pseudo-arrhenotoky^98–101^.

As a final note, our *C. arabica* PBDM can easily be modified to include other species of coffee (e.g., *C. robusta*), and has transferability enabling its use in a bio-economic analysis on larger, albeit global scale, and in the face of climate change.

## Supplementary information

Supplementary Information 1.
