# Supplementary material for: The coffee agroecosystem: bio-economic analysis of coffee berry borer control (Hypothenemus hampei)
Source: Sci Rep. 2020 Jul 23;10:12262. doi: 10.1038/s41598-020-68989-x (PMC7378549; doi:10.1038/s41598-020-68989-x)
Supplement: Supplementary file 1 — Supplementary Information 1. [file 41598_2020_68989_MOESM1_ESM.docx]

**Supplemental materials:**

**The coffee agroecosystem: bio-economic analysis of coffee berry borer control**

**(***Hypothenemus hampei***)**

José Ricardo Cure* ^1,4^, Daniel Rodríguez^1,4^, Andrew Paul Gutierrez^2,4^ & Luigi Ponti^3,4^

^1^ Facultad de Ciencias Básicas y Aplicadas, Universidad Militar Nueva Granada, Cr.11 No.101-80, Bogotá, Colombia

^2^ Division of Ecosystem Science, College of Natural Resources, University of California, Berkeley, CA 94720-3114, USA

^3^Agenzia nazionale per le nuove tecnologie, l’energia e lo sviluppo economico sostenibile (ENEA), Centro Ricerche Casaccia, Via Anguillarese 301, 00123 Roma, Italy.

^4^ Center for the Analysis of Sustainable Agricultural Systems Global (casasglobal.org), 37 Arlington Ave., Kensington, CA 94707, USA.

^*^corresponding author [jose.cure@unimilitar.edu.co](mailto:jose.cure@unimilitar.edu.co)

**A detailed overview of the coffee agroecosystem model used in the bioeconomic analysis**

To perform bioeconomic analyses of complex systems requires extensive field data that is not usually available. As an alternative, well parameterized models of the system^1,2,3,4^ may be used as the objective function in the analysis, and the simulation results analyzed using marginal analysis supplemented with field observations. This is the approach used in the bioeconomic analysis of the coffee/coffee berry borer/natural enemy system. Below we summarize the components of the coffee system model.

**The coffee system model**

The physiologically based demographic modeling (PBDM) approach to (agro) ecosystem analysis builds on the idea that all organisms (plants, herbivores, natural enemies and diseases) are consumers, and all have similar problems of resource acquisition (inputs) and allocation (outputs)^5^. This notion allows the use of the same resource acquisition model and birth-death dynamics models to describe all trophic levels including the economic one^6,7^. Specifically, consumers have time varying demands (*D*) in priority order for egestion, conversion costs, respiration (i.e., the Q10 rule in ectotherms), and reproduction, growth, and reserves. Organisms acquire the resource (i.e., the supply, *S*) by search. Plants seek to light, nutrients and water and search for them using leaves, roots or other mechanisms. Similarly, animals in all trophic levels search for prey/hosts. Because search is imperfect, the ratio 0 ≤ *S/D* ≤ 1 is always less than unity and scales maximal vital rates of species to the observed.

The main biotic elements and interrelations included in the coffee agroecosystem simulation model are in Gutierrez *et al.*^1^ and Rodriguez *et al.*^2,3,4^. Among the species is the coffee berry borer (CBB) that is attacked by four parasitoids, two fungal pathogens, and two parasitic nematodes. All species and their interactions in the simulation are driven by daily climatic variables (solar radiation, temperature, precipitation and relative humidity) and condition the behavior of all its components. The fungal diseases, nematodes and insecticides are applied as sprays to control CBB.

**Brief Overview of the Distributed-Maturation-Time Dynamics population sub-models**

(modified from Gutierrez and Ponti)^8^.

The Erlang distributed-maturation-time demographic model is widely used to simulate the age structured population dynamics of all species interacting in the system. Details concerning the time invariant and time varying models are found in Manetsch^9^, Vansickle^10^ and DiCola *et al.*^11^. The general form of the time invariant model for the *i*th age class of a population is as follows, equation (1).

$\frac{dN_{i}}{dt}=\frac{k\Delta_{x}}{\Delta}\left[N_{i-1}(t)-N_{i}(t)\right]-\mu_{i}(t)N_{i}(t)$ (1)

*N_i_* is the density (mass or numbers) of the *i*th age class, *dt* is the change in time (e.g. a day), *k* is the number of age classes, Δ is the expected mean developmental time, Δ*_x_* is the daily increment of physiological age (degree days) and *μ*_i_(*t*) is the proportional age-specific, net-loss rate as modified by temperature, age, net migration and mortality due to natural enemies (based on individual biology of the interacting species). The numerical solution of equation (1) outlined by Gutierrez^12^ using Euler’s integration is implemented in this work.

The coffee system model consists of 13 linked age structured sub-population models (according to equation (1): 7 for the plant, 3 for CBB and 4 for the parasitoids^2,3,4^. The plant model is a branch level time varying model consisting of sub-populations for the mass of leaves {*subpopulation* 1}, stems {2}, branches {3}, shoots {4} and roots {5}, and the mass and number of berries {6, 7}. The model for CBB on a per tree basis consists of age structured sub-populations for immature stages developing inside fruits {8}, dispersing adult females outside fruits {9} and females in colonized berries {10}. The parasitoids models consists of the sub-population models for *Cephalonomia stephanoderis* {11}*, C. hyalinnipennis*{12}*, Prorops nasuta*{13} and *Phymastichus cofeae*{14}.

The time step in all the dynamics models is a day of variable length in physiological time units (Δ*dd;* degree days computed using a nonlinear developmental rate model; see equation (14) below, as appropriate for each species and/or age. Cohorts of all populations have species average developmental times and variance. On average, a cohort completes development when

**First trophic level. The coffee plant model**

Summary based on Gutierrez *et al.*^1^and Rodriguez *et al.*^2^.

***Dynamics model***

Much of the important dynamics in the coffee plant occurs at the branch level, that consists of sub-populations for the mass of leaves {*subpopulation* 1}, stems {2}, branches {3}, shoots {4} and roots {5}, and the mass and number of berries {6, 7}.

***Canopy architecture and photosynthesis***

Leaves search for light to produce photosynthesis that is allocated to all subunit populations.

Under non limiting conditions, new branches are produced at a rate of one per 280*dda* (cumulative *dd*). However, the rate of new branch production slows with the level of the photosynthate supply/demand ratio that deviates from unity due to shortfalls in water and nutrients and other factors (linearly). The demand for photosynthate by the *j*th branch at time (*t*), *D_j_*(*t*), is calculated as in equation (2);

$D_{j}(t)=\frac{([D_{j,leaf}(t)+D_{j,stem}(t)+D_{j,fruit}(t)]/\lambda)+Q10}{\beta}$ (2)

where the branch demands of leaves (*D_j,leaf_*), stem (*D_j,stem_*) and fruit (*D_j,fruit_*), are corrected by *λ* (cost for converting resource to self), plus respiration costs *Q*10, and *β* (the proportion of acquired that is assimilated). The demand for the whole plant is computed by equation (3).

$D\left(t\right)\text{ }=\sum_{j=1}^{n(t)} D_{j}\left(t\right)\text{+ }D_{trunk}\left(t\right)\text{ }+\text{ }D_{root}\left(t\right)$ (3)

*D_trunk_* and *D_root_* are the contribution the branch makes to plant level trunk and root growth demands. Branches in different levels in the canopy intercept different amounts of light as proposed by Monsi and Saeki^13^. The proportion of incident solar radiation reaching to the different branch strata were described by the following equation (4).

, (4)

where *I_j_* is photosynthetic active radiation that reaches a particular strata (*n*) of the plant, *I_o_* is the amount of photosynthetically active radiation at the top of the canopy, *φ* is the light extinction coefficient, and *LAI_J_* is the leaf area index of branch *j*. The coefficient *φ* allows estimation of the proportion of light reaching branch level *j* estimated by equation (5)^14^.

(5)

The photosynthetic rate (supply of photosynthate) in branch strata *j*, *S_j_*, equation (6) is calculated using the type III Gutierrez-Baumgärtner functional response^1,14^.

$S_{j}=D_{j} \cdot L_{j}[1-\exp(\frac{-\alpha_{j}\cdot c I_{j}}{D_{j}\cdot L _{j}}]$ (6)

*D_j_* is the photosynthate demand of branch strata *j,* equation (2) (i.e. the amount required (g/g) in order to survive and keep growing), *L_j_* is the total dry mass of leaves converted to *LAI_j_* in branch level *j*. Coefficient *c* converts solar radiation *I_j_* ($cal\cdot cm^{-2}day^{-1}$) to potential g mass of potential photosynthate.

The lower thermal threshold for photosynthesis in *C. arabica* is 10°C and the optimal temperatures for photosynthesis in the range 20–25 ^o^C ^15,16^. This relationship is captured by a concave function that increases from 10°C and asymptotes at 20°C (in degree days, *Δdd* = *T(t)* -10^o^C). To capture the effects of temperature, a scalar function is used, equation (7)^2^.

(7)

scales S*_j_* to the realized photosynthetic rate ($\hat{S}_{j}(t,T)$), equation (8).

(8)

The effect of chilling on leaf area loss reported by Batista *et al.*^16^ is simulated dynamically through mass attrition of older leaves on each branch at the rate .

***Fruiting phenology and dynamics***

Induction of flower buds at maturity in coffee is inversely related to solar radiation (*I_o_*), with the highest rates occurring at 9 MJ∙m^−2^∙day^−1^ and declines to zero at 13 MJ m^−2^ day^−1^ (see Drinnan^17^). Ignoring *t* for simplicity, the number of latent buds induced on branch *j* day^−1^ is computed by equation (9)^2^,

$\Delta buds_{j}=9.57wood_{j}\times(2.87\times10^{-3}\text{- 4.1}\times10^{-6}\times\text{I}_{\text{o}}\text{)}\times\Delta\text{dd }$ (9)

where *wood_j_* is the stem mass accumulated in the *j* branch in the previous season. The new buds (stage 1) enter the branch level fruit dynamics model at age zero, and develop for an additional 75 days (∼840 *dda*)^18^ at which time they enter dormancy^17,19,20,21,22,23^. Latent stage 1 buds accumulate on the branches over time during dry periods and may remain dormant as stage 2 buds for up to 4 months (840–2562 *dda*)^24^. However, when the rains begin, the buds break dormancy at a rate (*p*) proportional to the intensity of the dry period preceding the rains as measured by the hydric potential $\psi_{L}$of the leaves. We use the logistic function of Drinann^17^, equation (10), to describe the proportion (*p*) of latent buds that flower at time *t*, with *a* and *b* being constants estimated for each locality.

$p=\frac{1}{1+e^{a+b\psi_{L}}}$ (10)

$\psi_{L}$ is a function of the hydric potential of the leaves and was calculated from the number of days with very low or not rain, using a quadratic equation (11) fitted to the data reported by Drinann^2,17^; units are in megapascals (MPa),

$\psi_{L}=-0.764091+0.257113d_{dry}-0.0395d_{dry}^{2}$ (11)

The number of buds that switch to flowering was estimated by multiplying *p*, equation (10), by the number of latent buds at time *t*, equation (9). The buds induced to flower in each node enters a population array for that branch, and are followed through their transit of growth and development in physiological time units until harvest or natural dehiscing (i.e. using the model of Vansickle^10^). The maximum number of buds/nodes that may break dormancy during each dormancy-terminating episode is 12.

***Water dynamics***

Ritchie^25^ model was used to calculate water demand of the crop, its transpiration and soil evaporation. As in Gutierrez *et al.*^1^, plant demands for water use as function of temperature, incident radiation and *LAI* are simulated. Water uptake by the roots is modeled using a functional response model, equation (12).

$S_{w}=D_{w}(1-\exp(\frac{-\alpha_{w}W}{D_{w}}))$ (12)

Where $S_{w}$ is the daily water supply, $D_{w}$ is the daily water demand, $W$is the water available in the soil to the plant ($W$= *field capacity* – *permanent wilting point*) and *α_w_* is a measure of the efficiency of the roots for water uptake.

***Nitrogen dynamics***

For nitrogen dynamics we used the routine reported by Gutierrez *et al.*^14^ that performs daily balance of the nitrogen in the volume of soil explored by the roots of the plant. As in Gutierrez *et al.*^14^, we assume the same rates of nitrogen mineralization of organic matter and nitrogenous fertilizer applications. An estimated 50% of nitrogen is assumed lost due to water flow through and volatilization. The time of nitrogen fertilization used in the simulations was based on Sadeghian^26^. Demands of nitrogen by the different plant organs were based on reports of nitrogen content in dry matter accumulation under proper fertilization management^27,28^. A functional response model similar to equation (12) is used for nitrogen uptake where the demand for nitrogen is proportional to the demand for photosynthate.

***Effects of multiple resources***

The compounding effects of the water and nitrogen supply/demand ratios are scalars that are similar to the effects of temperature and included in the model () (i.e. $\hat{S}_{j}(T,N,W)=\varphi_{T}\varphi_{W}\varphi_{N}\cdot Sj(t)$; see equation (8)^12^

**The Second trophic level – the CBB model**

Summary based on Gutierrez *et al.*^1^ and Rodriguez *et al.*^3^.

***The dynamic sub-models***

CBB dynamics is built upon the sub-populations of mass and number of berries {6, 7}, see equation (1). CBB sub-populations, on a per tree basis, consists of three age structured sub-populations: immature stages developing inside fruits {*subpopulation* 8}, dispersing adult females outside fruits {9} and females in colonized berries {10}. The distributed maturation time population dynamics model simulates the observed dispersion in maturation times of cohorts of individuals^29^. A constant immigration rate of 0.25 CBB females per day and plant was used in the model.

Data from Jaramillo *et al.*^30^ were used to estimate the CBB developmental rates *R(T(t))* from egg to adult at different temperatures. The simplest model is when the temperature is in the linear range of development, equation (13) with parameters *a* and *b*.

$R(T(t))=a+bT(t)$ (13)

Solving for *R(T(t)) = 0* yields the threshold *θ* (from equation (2), $\theta=-a/b$), and hence the developmental time in degree days (*dda*) for each life stage (stage subscript ignored) can be computed as $\Delta=(T-\theta)/R(T)$However, development is not linear, and to make the model more general, we used the model proposed by Lactin *et al.*^31^ to estimate *R(T(t))*, equation (14)*.*

$R(T(t))=\lambda+\exp(\rho T(t))-\exp\left[\rho T_{m}-\frac{T_{m}-T(t)}{T_{u}}\right]$ (14)

$\lambda$makes the curve intercept the x-axis, allowing the estimation of the minimum thermal threshold (see Table 1), $\rho$ can be interpreted biologically as a compounded $Q_{10}$ being the resultant of critical enzymatic reactions in the process of development, $T_{m}$ is the maximum temperature threshold, and *T_u_* is the approximate amplitude of the range where high temperatures produce negative effects on the physiology of the organism^32^. Equation (14) may be viewed as the proportion of the development completed at temperature *T* at time t, with completion of the stage occurring when $\int R(T(t))dt=1$.

***Resource Acquisition***

The total number of berries available for attack,$Bd(t)$, equation (15), is the sum of berries in each age class *a*, at time *t* (i.e., $B(t,a)$) corrected for preference $\vartheta(a)$^1^.

$Bd(t)=\sum_{0}^{a_{MAX}} \vartheta(a)B(t,a)da$ (15)

In the field, a very small proportion of multiple attacks per berry (super parasitism) are observed^33,34^, as one female per berry is the general case. Hence, if $N_{b}(t)$ is the number of searching females, the demand for unattacked fruits is also $N_{b}(t)$. We use the Fraser and Gilbert^35^ functional response model^12^, equation (16), which accounts for super parasitism, where the number of newly attacked berries at time *t* is $S_{b}(t)$ and $\alpha_{b}$is the female search.

$\text{S}_{\text{b}}\text{(t)=Bd(t)[1-exp(}\frac{\text{-N}_{\text{b}}\text{(t)(1-exp(}\frac{\text{-α}_{\text{b}}\text{(t)Bd(t)}}{\text{N}_{\text{b}}\text{(t)}}\text{))}}{\text{Bd(t)}}\text{)]}$ (16)

Search is imperfect, and hence the supply/demand ratio is the proportion of searching females finding new berries to colonize^12^, and estimates adult mortality to the local population due emigration from the female population. The numbers of healthy and attacked berries are followed in the model by means of separate age structured arrays^1^.

***Mortality factors***

In the case of CBB, the $\mu_{i}(t)$ parameter in equation (1) is the sum of the partial components of mortality age specific proportional net loss rate due to all causes outlined below and affect the dynamics of all stages: $\mu_{R}$is rain mortality, $\mu_{imo}$is the intrinsic mortality, $\mu_{comp}$ is due to intraspecific competition, $\mu_{traps}$is mortality of searching females in baited traps, $\mu_{ef}$ is mortality to the population due to emigrating females that fail find berries to colonize, and $\mu_{imm}$is the immigration rate (i.e., a positive inflow, Gutierrez^12^; p. 145). Specifically, $\mu_{i}$ for eggs, pupae, pre-ovipositing adults, and females that successful colonize new berries is

$\mu_{i}=1-{(1-\mu}_{R})(1-\mu_{imo})$, for larvae $\mu_{i}={1-(1-\mu}_{R})(1-\mu_{imo})(1-\mu_{comp}$) and for searching adult females outside of berries $\mu_{i}=1-(1-\mu_{R})(1-\mu_{imo})(1-\mu_{traps})(1-\mu_{ef})(1+\mu_{imm})$

# The model by Nuessly *et al.*^36^ to estimate mortality in *Heliothis zea* due to rainfall was used for adult and immature survivorship of CBB, equation (17).

$\mu_{R}=1-e^{-\beta R}$ (17)

Mortality $\mu_{R}$is estimated in function of rain, *R*, expressed in millimeters and *β* is an empirical coefficient estimating mortality rate due to precipitation.

Intraspecific competition is assumed to have two components: reduction in fecundity^37^ and increases in larval mortality^38^. Data to estimate these effects were not available and hence we assumed that a reduction in fecundity *F*(*t*), equation (18), begins above a low threshold density of *U_1_*=4 larvae per berry and the increases in mortality *μ(t)_comp_* , equation (19) at a threshold density of *U_2_*=4 larvae per berry for larvae and adults and *U_2_*= 5 larvae per berry for pupae. These values were estimated recursively using the simulation model to search for the value of *U* that best reproduced the mean number of observed eggs and larvae per fruit across all data sets.

$F=\left\{\begin{matrix} \begin{matrix} \frac{F_{\max}}{1+\left(LR-U_{1}\right)} for LR>U_{1} \\ \end{matrix} \\ F_{\max} otherwise \end{matrix}\right\}$ (18)

where *F* is the realized fecundity rate per female, *F*_max_ the potential maximum fecundity rate under optimal conditions, and *LR* the ratio of total larvae on total fruits attacked in the tree.

$\mu_{comp}=\left\{\begin{matrix} \mu_{o}\cdot(1+LR-U_{2}) for LR>U_{2} \\ \mu_{0} otherwise \end{matrix}\right\}$ (19)

$\mu_{0}$ is the intrinsic mortality rate for larvae under optimal conditions absent intraspecific competition (see Romero & Cortina^39^).

Gil *et al.*^40^, equation (20), evaluated the recaptures of CBB adults in pheromone Brocatrap® design traps at several distances from a coffee crop. These data were used to simulate the capture mortality of seeking females,

$p=0.17719-0.040457\cdot\text{log}_{e}(d)$ (20)

where *p* is the proportion of females captured and *d* is the radius that depends on the density of traps per unit area. In the model we used *d*=16.3.

Precipitation and temperature affect the emergence of available new adults in our age structured model. We use a variable width emergence window that expands with the favorability of environmental conditions. Specifically, when temperature decrease and the precipitation increase, the window contracts). When adverse conditions remain unfavorable for long periods, new females do not emerge and reproduce in their host berry^41,42^. Once reproduction occurs, the wings of females atrophy, and they are no longer able to leave the berry^43^. In order to simulate this behaviour, it is assumed that individuals that reach the half of the time as adult (544.73 *dd*) will not emerge and will reproduce in the native berry^3^. This biology is captured by equation (21),

$E=312.47\times\frac{1+P\cdot0.1}{\mu_{ddaCBB}\cdot0.185}$ for $312.47<E<544.73$ (21)

where *E* is the emergence time of CBB females in degree days, *P* is the daily precipitation in mm, $\mu_{ddaCBB}$ is the mean of degree days for CBB in the last three days, and 312.47 is the minimum age of adult emergence in degree days. This equation was calibrated via simulation.

CBB mortality due to parasitoid attack on adults or larvae inside fruits or in adults tunneling in the berry were also incorporated in the models as explain in the next section. Mortality from chemical and/or biological sprays were explained in the main text of this publication.

**Third trophic level. CBB parasitoids**

Although natural enemies are not effective in regulation CBB, we included them here for completeness. This summary is based on Gutierrez *et al.*^1^ and Rodriguez *et al.*^4^.

The parasitoids model consists of the population submodels for *Cephalonomia stephanoderis* {11}, *Cephalonomia hyalinipennis*{12}, *Prorops nasuta* {13} and *Phymastichus coffea* {14}. The developmental biology of each species and the linkages of between them and their trophic interactions are illustrated in Fig. 1 (main text of this publication). Eggs oviposited by parasitoid females enters to population submodels {11}, {12}, {13} and {14} as $N_{0}(t)$; see equation (1).

***Bethylid parasitoids*** - A bethylid female (e.g. *Cephalonomia hyalinipennis, C.stephanoderis* and *P.nasuta*) enter only one CBB infested berry where they attack the CBB life stages and those of competing parasitoid species. Colonization by bethylid parasitoids is a two-stage process: the 1^st^ stage involves finding a CBB infested berries and the 2^nd^ stage occurs within the berry when the parasitoid female attacks CBB life stages either for host-feeding or parasitism. Both stages are estimated via the predator form of the Gutierrez-Bäumgartner functional response model^12^, equation (22).

$S_{k}(t)=D_{k}(t)A_{k}(t)(1-\exp(-\frac{\alpha_{k}B_{a,k}(t)}{D_{k}(t)A_{k}(t)})$ (22)

In the 1^st^ stage of attack, *S_k_ (t)* is the number of CBB infested berries colonized by the *k^th^* bethylid species, *B_a,k_ (t)* is the number of infested berries with different CBB biological stages suitable for parasitization and host-feeding by the population of the *k^th^* species of betilid females (*A_k_ (t)*), *α_k_* is the parasitoid search rate and *D_k_ (t)* is the parasitoid demand for CBB infested berries (i.e. 1 per parasitoid female). *C. hyalinipennis* requires a longer time to colonize a berry than the other bethylids^44^ and this attack biology is reflected in the lower search rate $\alpha_{k}$. Specifically, $\alpha_{k}$for *C. hyalinipennis* is 0.5, and that for *C. stephanoderis* and *P. nasuta* is 0.9.

In the 2^nd^ stage within the berries, the same functional response, equation (22) is used to capture the biology of the betylid species attack on CBB life stages. *S_k_ (t)* is the number of immature available for parasitization by the *k^th^* parasitoid *A_k_* *(t)* is the population of the female of the *k^th^* parasitoid attacking CBB stages, *B_a,k_ (t)* is the number of CBB available for parasitism or host-feeding corrected for preference, $\alpha_{k}=1$ is the search rate of bethylids inside the berry, and *D_k_ (t)* is the temperature dependent per-capita demand for parasitism and host-feeding. *C. hyalinipennis* super parasitizes conspecifics and the other bethylid species and lays 1 to 3 eggs per host^45^.

***Competition among bethylids***

Competition between individual parasitoids of two different species (dyadic contest) occurs in coffee berries when a bethylid female enters a berry previously colonized by a female of another species^44^. This behavior is more likely to occur when CBB infested berries are scarce. In the model, dyadic contest occurs at a rate dependent on the shortfall of CBB infested berries attacked (i.e.$1-[S(t)/D(t)] >0$) (see Rodriguez *et al.*^4^). The different success rates are included in the model via the parameter $\delta$, which weights the proportion of total contests won by each parasitoid in dyadic contests (Rodriguez *et al.*^4^, modified from Batchelor *et al.*^44^). When a second species of a bethylid parasitoid enters a berry previously colonized by another parasitoid species, it attacks the immature stages of both CBB and the first parasitoid. Facultative oviposition and host-feeding interactions between bethylid species was also included^4^.

***Phymastichus coffea*** - The Fraser and Gilbert^35^ parasitoid functional response model, equation (23) is used to calculate the parasitism rates by *P. coffea* females which may attack more than one CBB and because it allows super parasitism (see Gutierrez^12^).

$S_{Pc}(t)=B_{a}(t)\left[(\right.1-\exp\frac{-D_{Pc}(t)A_{Pc}(t)(1-\exp(\frac{-\alpha_{Pc}(t)B_{a,Pc}(t)}{D_{Pc}(t)A_{Pc}(t)}))}{B_{a,Pc}(t)}\left.)\right]$ (23)

*S_Pc_* is the number of CBB females parasitized at time *t*, *B_a,Pc_(t)* is the number of CBB females attacking berries corrected for preference that varies with maturation stage of the berry^46^. Specifically, *P. coffea* preference for CBB females attacking stage 1 = 0.0, stage 2 = 0.6, stage 3 = 0.8 and stage 4 = 0.31. The parameter $\alpha_{Pc}$ is the search rate of the parasitoid, *D_Pc_(t)* is the per capita demand for hosts per female per *dd* at time *t*, and *A_Pc_(t)* is the number of adult parasitoid females seeking CBB females. The ratio $0\leq S_{Pc}(t)/[D_{Pc}(t)A_{Pc}(t)]<1$ is an estimate of *P. coffea* parasitization success^12^. Direct competition occurs only among the betilids species, while indirect competition occurs with *P. coffea*.

**Bibliography**

1. Gutierrez, A.P., Villacorta, A., Cure, J.R. & Ellis, C.K. Tritrophic analysis of the coffee (*Coffea arabica*)-coffee berry borer (Hypothenemus hampei (Ferrari))-parasitoid system. *Anais da Sociedade Entomológica do Brasil* **27(3),** 357-385 (1998).

2. Rodríguez, D., Cure, J.R., Cotes, J.M., Gutierrez, A.P. & Cantor, F.A. coffee agroecosystem model I. Growth and development of the coffee plant. *Ecological Modelling* **222(19),** 3626–3639 (2011).

3. Rodríguez D, Cure J.R., Cotes J.M., Gutierrez A.P. & Cantor, F. A coffee agroecosystem model: II. Dynamics of coffee berry borer. *Ecological Modelling* **248,** 203–214 (2013).

4. Rodríguez, D., Cure, J.R., Cotes, J.M. & Gutierrez, A.P. A coffee agroecosystem model. III. Parasitoids of the coffee berry borer (Hypothenemus hampei). *Ecological Modeling* **363,** 96-110 (2017).

5. Gutierrez, A.P. Physiological basis of ratio-dependent predator–prey theory: the metabolic pool model as a paradigm. *Ecology* **73,** 1552–1563 (1992).

6. Regev, U., Gutierrez, A.P., Schreiber, S.J. & Zilberman, D. Biological and economic foundations of renewable resource exploitation. *Ecological Economics* **26,** 227-242 (1998).

7. Gutierrez, A.P. & Regev, U. The bioeconomics of tritrophic systems: applications to invasive species. *Ecological Economics* **52,** 382-396 (2005).

8. Gutierrez, A.P. & Ponti, L. Assessing the invasive potential of the Mediterranean fruit fly in California and Italy. *Biol. Invasions* **13,** 2661–2676 (2011).

9. Manetsch, T.J. Time-varying distributed delays and their use in aggregate models of large systems. *IEEE Trans.* *Syst. Man Cybern.* **6,** 547–553 (1976).

10. Vansickle, J. Attrition in distributed delay models. *IEEE Trans Syst Man Cyber*n 7: 635–638 (1977).

11. DiCola, G., Gilioli, G. & Baumgärtner, J. Mathematical models for age-structured population dynamics in *Ecological Entomology* (ed. Huffaker, C.B., Gutierrez, A.P.) 503–534 (John Wiley and Sons, New York, 1999).

12. Gutierrez, A.P. *Applied Population Ecology: A Supply–Demand Approach* 1-320 (John Wiley & Sons, United States, 1996).

13. Monsi, M. & Saeki, T. Über den lichtfaktor in den pflanzengesellschaften und seine bedeutung für die stoffproduktion. *Jpn. J. Bot.* **14,** 22–52 (1953).

14. Gutierrez, A.P. *et al.* A model for the growth and development of three varieties of common bean (Phaseolus vulgaris L.): factors affecting yield and quality. *Agric. Syst.* **44,** 35-63 (1994).

15. Mosquera, L., Riaño, N., López, Y. & Arcila, J. Net photosynthesis and CO2 compensation concentration in three coffee *(*Coffea sp.) genotypes, bean and maize under three temperatures. *Revista Facultad Nacional de Agronomía*, Medellín (Colombia) **58,** 2827-2835, <https://revistas.unal.edu.co/index.php/refame/article/view/24243> (2005).

16. Batista, S.P. *et al.* The impact of cold on photosynthesis in genotypes of Coffea spp – photosystem sensitivity, photoprotective mechanisms and gene expression. *J. Plant. Phys.* **168,** 792–806 (2011).

17. Drinnan, J. *The control of floral development in coffee (Coffea arabica L.)*. PhD. Dissertation. 1-157 (University of Queensland, Australia, 1992).

18. Arcila, J., Buhr, L., Bleiholder, H., Hack, H. & Wicke, H. Aplicación de la “Escala BBCH Ampliada” para la descripción de las fases fenológicas del desarrollo de la planta de café (Coffea sp.). Centro Nacional de Investigaciones del café “Pedro Uribe Mejía”, Colombia, 1-32 <http://biblioteca.cenicafe.org/bitstream/10778/578/3/bot0023.pdf>, (2001).

19. Mes, M.G. Studies on the flowering of Coffea arabica L.: I. The influence of temperature on the initiation and growth of coffee flowering buds. *Portugaliae Acta Biologica* **4,** 328-341 (1957).

20. Pagaez, E.A. Quelques considerations sur la floraison du cafeier. *Bull Agric Congo Belge* **50,** 1531-1540 (1959).

21. Frederico, D. & Maestri, M. Ciclo de crescimento dos botões florais do café. *Revista Ceres* Universidade Federal de Viçosa (Brazil) **17,** 171–182 (1970).

22. Gopal, N.H. & Vishveshwara, S. Flowering of coffee under South Indian conditions. *Indian Coffee* **35,** 142-154, <http://opac.bibliotecaorton.catie.ac.cr/cgi-bin/koha/opac-detail.pl?biblionumber=426824> (1971).

23. Da Matta, F.M., Ronchi, C.P., Maestri, M. & Barros, R.S. Ecophysiology of coffee growth and production. *Bras. J. Plant. Phys.* **19,** 485–510 (2007).

24. Cannell, M.G.R. Physiology of the coffee crop, in *Coffee Botany, Biochemistry and Production of Beans and Beverage* (eds. Clifford, M.N., Willson, K.C.) 1-480 (Springer, USA, 1985).

25. Ritchie, J.T. Model for predicting evaporation from a row crop with incomplete cover. *Water Resour. Res.* **3,** 1204-1213 (1972).

26. Sadeghian, S. *Fertilidad del Suelo y Nutrición del Café en Colombia*. 1-44, <https://www.cenicafe.org/es/publications/bot032.pdf> (Centro Nacional de Investigaciones del café “Pedro Uribe Mejía”, Chinchiná, Colombia, 2008).

27. Riaño, N., Arcila, J., Jaramillo, A. & Chaves, B. Acumulación de materia seca y extracción de nutrimentos por Coffea arabica L. cv. Colombia en tres localidades de la zona cafetera central. *Revista Cenicafé* (Colombia) **55,** 265–276, <https://www.cenicafe.org/es/publications/arc055%2804%29265-276.pdf> (2004).

28. Harmand, J.M. *et al.* Nitrogen dynamics and soil nitrate retention in a Coffea arabica-Eucalyptus deglupta agroforestry system in Southern Costa Rica. *Biogeochemistry* **85(2),** 125–139 (2007).

29. Severini, M., Baumgärtner, J. & Limonta, L. Parameter estimation for distributed delay based population models from laboratory data: egg hatching of Oulema duftschmidi Redthenbacher (Coleoptera: Chrysomelidae). *Ecological Modeling* **167,** 233–246 (2003).

30. Jaramillo, J. *et al.* Thermal tolerance of the coffee berry borer Hypothenemus hampei: Predictions of climate change impact on a tropical insect pest.  PLoS ONE 4(8): e6487; <https://doi.org/10.1371/journal.pone.0006487> (2009).

31. Lactin, D.J., Holliday, N.J., Johnson, D.L. & Craigen, R. Improved rate model of temperature dependent development by arthropods. *Environmental Entomology* **24(1),** 68–75 (1995).

32. Sigsgaard, L. The temperature-dependent duration of development and parasitism of three cereal aphid parasitoids, Aphidius ervi, A. rhopalosiphi and Praon volucre. *Entomologia Experimentalis et Applicata* **95(2),** 173–184 (2000).

33. Borsa, P. & Gingerich, D.P. Allozyme variation and an estimate of the inbreeding coefficient in the coffee berry borer Hypothenemus hampei (Coleoptera: Scolytidae). *Bulletin of Entomological Research* **85(1),** 21–28 (1995).

34. Benavides, P. Aspectos genéticos de la broca del Café in *La broca del café en América Tropical: Hallazgos y Enfoques*. (eds. Barrera, J.F., García, A., Domínguez, V., Luna, C.), 101–110, <http://www2.tap-ecosur.edu.mx/mip/pdf/BrocadelCafeHallazgosyEnfoques2007.pdf> (Sociedad Mexicana de Entomología and El Colegio de la Frontera Sur, México, 2007).

35. Fraser, B.D. & Gilbert, N. Coccinelids and aphids: a quantitative study of the impact of adult ladybirds (Coleoptera:Coccinelidae) preying on field populations of pea aphids (Homoptera: Aphididae). *Journal of the Entomological Society of British Columbia* **73,** 33–56 (1976).

36. Nuessly, G.S., Hartstack, A.W., Witz, J.A. & Sterling, W.L. Dislodgement of Heliothis zea (Lepidoptera: Noctuidae) eggs from cotton due to rain and wind: A predictive model. *Ecological Modelling* **55(1-2),**  89–102 (1991).

37. Vega, F.E., Kramer, M. & Jaramillo, J. Increasing coffee berry borer (Coleoptera: Curculionidae: Scolytinae) female density in artificial diet decreases fecundity*. Journal of Economic Entomology* **104 (1),** 87–93 (2011).

38. Baker, P.S., Barrera, J.F. & Rivas A. Life-history studies of the coffee berry borer (Hypothenemus hampei Scolytidae). *Journal of Applied Ecology* **29(3),** 656–662 (1992).

39. Romero, J.V. & Cortina, H.A. Tablas de vida de Hypothenemus hampei (Coleoptera: Curculionidae: Scolytinae) sobre tres introducciones de café. *Rev. Colomb. Entomol.* **33(1),** 10–16, <http://www.scielo.org.co/pdf/rcen/v33n1/v33n1a02.pdf> (2007).

40. Gil, P., Zulma, N., Acevedo, B.F.E., Benavides, P. & Bustillo, A.E. Radio de acción de la trampa atrayente Brocatrap® para la captura de adultos en un cafetal in *Resúmenes XXXIV Congreso de Entomología, Sociedad Colombiana de Entomología. Cartagena de Indias, Colombia No.199,* 129, <https://repository.agrosavia.co/handle/20.500.12324/34389> (ed. González, P.H. & Hernández, D.J., 2007).

41. Bustillo, A.E. Una revisión sobre la broca del café, Hypothenemus hampei, en Colombia. *Rev. Colomb. Entomol.* **32(2),** 101-116, <http://www.scielo.org.co/pdf/rcen/v32n2/v32n2a01.pdf> (2006).

42. Bustillo, A.E. *El manejo de cafetales y su relación con el control de la broca del café en Colombia.*1-40, <http://biblioteca.cenicafe.org/bitstream/10778/579/1/024.pdf> (2nd Ed. Chinchiná, Colombia: Cenicafé, 2007).

43. Ticheler, J.H.G. Etude analytique de l’epidemiologie du scolyte des grains de cafe, Stephanoderes hampei Ferr en Cote d’Ivoire, *Meded. Land-bouwhogeschool*, Wageningen, **6,** 1–49. <https://edepot.wur.nl/290697> (1961).

44. Batchelor, T.P., Hardy, I.C.W., Barrera, J.F. & Pérez-Lachaud, G. Insect Gladiators II: Competitive interactions within and between parasitoids species of the coffee berry borer Hypothenemus hampei (Coleoptera: Scolytidae). *Biological Control* **33,** 194-202 (2005).

45. Pérez-Lachaud, G. & Hardy, I.C.W. Reproductive biology of Cephalonomia hyalinipenis (Hymenoptera:Bethylidae) a native parasitoid of the coffee berryborer, Hypothenemus hampei (Coleoptera: Scolytidae) in Chiapas, México. *Biological Control* **14,** 152–158, (1999).

46. Jaramillo, J., Bustillo, A.E. & Montoya, E.C. Parasitismo de Phymastichus coffea sobre poblaciones de Hypothenemus hampei en frutos de café de diferentes edades. *Revista Cenicafé* (Colombia) **53,** 317–326, <https://www.cenicafe.org/es/publications/arc053%2804%29317-326.pdf> (2002).
